# The Effect of Concentration of Lithium Salt on the Structural and Transport Properties of Ionic Liquid-Based Electrolytes

**DOI:** 10.3389/fchem.2019.00945

**Published:** 2020-02-04

**Authors:** Jiahuan Tong, Shengli Wu, Nicolas von Solms, Xiaodong Liang, Feng Huo, Qing Zhou, Hongyan He, Suojiang Zhang

**Affiliations:** ^1^Beijing Key Laboratory of Ionic Liquids Clean Process, Institute of Process Engineering, Chinese Academy of Sciences, Beijing, China; ^2^Department of Chemical & Biochemical Engineering, Technical University of Denmark, Lyngby, Denmark; ^3^College of Chemistry and Chemical Engineering, University of Chinese Academy of Sciences, Beijing, China

**Keywords:** ionic liquids, electrolytes, battery, structure and property, MD simulations

## Abstract

Ionic liquids (ILs) are used as electrolytes in high-performance lithium-ion batteries, which can effectively improve battery safety and energy storage capacity. All atom molecular dynamics simulation and experiment were combined to investigate the effect of the concentration of lithium salt on the performance of electrolytes of four IL solvents ([C_*n*_mim][TFSI] and [C_*n*_mim][FSI], *n* = 2, 4). The IL electrolytes exhibit higher density and viscosity; meanwhile, larger lithium ion transfer numbers as the concentration of LiTFSI increases. Furthermore, in order to explore the effect of the concentration of lithium salt on the ionic associations of Li^+^ and anion of IL, the microstructures of the lithium salt in various IL electrolytes at different concentrations were investigated. The structural analysis indicated that strong bidentate and monodentate coordination was found between Li^+^ and anion of all IL electrolytes. Both cis and trans isomerism of [FSI]^−^ were observed in [FSI]^−^-type IL electrolyte systems. Furthermore, the existence of the ion cluster [Li[anion]_*x*_]^(x−1)−^ in IL electrolytes and the cluster became more closed and compact as the concentration of LiTFSI increases.

## Introduction

With the popularity of personal portable electronic devices, new energy vehicles and renewable energy are developing rapidly. The electrochemical energy storage system with high energy density, high cycle stability, and high power density is facing enormous challenges, and has gradually become the main research direction in the world. Lithium ion batteries have dominated the battery market since their successful commercialization in the early 1990s due to their high voltage, high specific energy, and long cycle life (Scrosati and Garche, 2010; Goodenough and Kim, 2011). However, battery performance and composition requirements are becoming more and more stringent as the application requirements continue to improve. The concern of the safety for lithium ion battery has been exposed and increased prominent, as it is difficult to meet the requirements of lightweight, high-capacity, long-life electronic equipment, electric vehicles, and other technologies. Therefore, the development of a new generation of green battery systems with high performance and environmental protection has become a common challenge for the international community.

Electrolyte, as a key component of lithium battery, not only plays a role in conducting lithium ions and conducting internal circuit, but also is one of the most important factors that determine battery capacity and cycle stability. Excellent battery electrolyte generally has the following characteristics: (1) good chemical and electrochemical stability, i.e., not reacting with the electrode in the operating voltage range; (2) high lithium ion transport capacity; (3) good compatibility with positive electrode and lithium metal negative electrode; (4) excellent electronic insulation performance; (5) low cost, low toxicity, and environmental protection, etc. However, the most widely used organic solvent electrolyte in industry cannot meet all the above comprehensive performance currently. Therefore, optimization and design of electrolyte composition and formula has become one of the best ways to promote the rapid development of lithium ion batteries (Xu, 2014; Kim et al., 2015; Wu et al., 2019).

**Figure 1:**
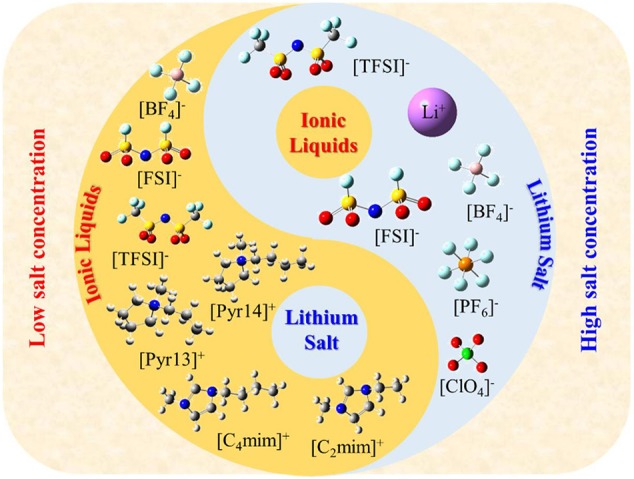
The common structure of ionic liquids and lithium salt used in lithium ion batteries (ionic liquids wrap lithium salt when the concentration of lithium salt is low; on the contrary, ionic liquids are wrapped with lithium salt).

Ionic liquids (ILs) are defined as molten salts with a melting point below 100°C, which is considered to be the third type of solvent after water and organic solvents. Meanwhile ILs have unique properties, such as high thermal stability, negligible vapor pressure, non-volatility, high ionic conductivity, etc. (Galinski et al., 2006; Lewandowski and Swiderska-Mocek, 2009; Zhang et al., 2018). Therefore, they are widely used in many energy storage devices, such as electrochemical double-layer capacitors, solar cells, etc., especially as electrolytes in lithium batteries in recent years (Figure S1). However, these properties are much affected by Coulombic interactions, van der Waals interactions, and the directionality of interactions between cations and anions. Thus, we should carefully consider these characteristics and structures as well as select the appropriate ILs in practical applications. Due to the flexibility of IL structure (theoretically, there are 10^18^ types of ILs), it is impossible to verify all ILs through experiments and select the best system for lithium ion batteries. Up to now, in the application of lithium batteries, only a few ILs are used as electrolytes (Figure 1). Meanwhile, this figure also indicates that the structure of IL electrolytes changes with the concentration of lithium salt; that is, the lithium salt is wrapped by IL when the concentration of lithium salt is low; on the contrary, the ILs are wrapped by lithium salt when the concentration of lithium salt is high. Therefore, in this study, we first summarize the recent progress in the field of IL electrolyte and aim to shed light on the future roadmap of this area of research.

Since the commercialization of lithium batteries, the electrolytes are based on organic carbonate. Although these types of electrolytes allow large numbers of charge and discharge cycles, there are some serious safety issues due to flammability and volatility, etc. In the past decades, lots of efforts have been paid to solve this issue to find potential alternatives to available solvent. Due to the enormous advantages of ILs, a common strategy is to test available ILs to take the place of problematic organic carbonates, which has been widely applied. Sakaebe and Matsumoto (2003) found that Li/LiCoO_2_ cell containing *N*-methyl-*N*-propylpiperidinium bis(trifluoromethanesulfonyl)imide as electrolyte showed good performance with a consistent capacity of LiCoO_2_ and a Coulombic efficiency at entire cycles of more than 97% at C/10 current rate. After that, Garcia et al. (2004) investigated ethyl-methyl-imidazolium bis-(trifluoromethanesulfonyl)-imide ([C_2_mim][TFSI]) doped with LiTFSI as electrolyte in lithium battery. The results were compared with conventional liquid organic solvent ethylene carbonate/carbonates electrolytes (EC/DMC). They found that IL electrolytes could provide better cycling performance; meanwhile, the conductivity was similar to that of the organic solvent electrolyte, reaching 7 mS/cm. Gradually, more researchers found that IL electrolytes not only could effectively improve the conductivity but also shows its advantages in the stability and cyclicity of the electrode and battery, respectively. For example, Ishikawa et al. (2006) first reported pure IL 1-ethyl-3-methylimidazolium bis(fluorosulfonyl)imide ([C_2_mim][FSI]) and N-methyl-N-propylpyrrolidinium bis(fluorosulfonyl)imide ([Pry13][FSI]) as electrolyte could provide a stable and reversible capacity for a graphitized negative electrode without any additives or solvents at ambient temperature. Experimental results also found that the reversible capacity of a graphite negative electrode has a stable value of ~360 mAh/g during 30 cycles at a charge/discharge rate of 0.2 C. Subsequently, Sugimoto et al. (2010) compared IL electrolytes [C_2_mim][FSI] and DMC in silicon–nickel–carbon composite anode for rechargeable lithium ion batteries. The experimental results showed that galvanostatic cycling of the Si-based composite anode in the FSI^−^-based electrolyte with a charge limitation of 800 mAh/g was stable and provided a discharge capacity of 790 mAh/g at the 50th cycle. At the same time, more and more researchers realized that IL electrolytes could be used for various electrodes in lithium battery with high performances (Chagnes et al., 2005; Kim et al., 2008; Zhang et al., 2008; Ma et al., 2019).

However, only from the experimental research on IL are electrolytes very limited because ILs are too complicated in comparison with common solvents. Therefore, it has been widely developed to explore the microstructures and properties of electrolyte by simulation methods. Borodin et al. investigated Li^+^ cation environment, transport, and mechanical properties for N-methyl-N-propylpyrrolidinium bis(trifluoromethanesulfonyl) imide ([mppy][TFSI]) and *N, N*-dimethyl-pyrrolidinium bis(trifluoromethanesulfonyl)imide ([mmpy][TFSI]) IL with 0.25 mol/L LiTFSI salt at 303–500 K by molecular dynamics simulations. The result revealed that <4 oxygen atoms coordinated with Li^+^ cation on average; meanwhile, the ion self-diffusion coefficients followed the order Li^+^ < TFSI^−^ < mmpy^+^ or mppy^+^ (Borodin et al., 2006). Recently, dynamical and structural properties of two IL electrolytes (LiTFSI-[C_2_mim][TFSI] and LiTFSI-[Pyr13][TFSI]) were investigated by Lesch et al. (2016) via all atom molecular dynamics simulations method. They found the cation of ILs was independent from the structure of the coordination shell of Li^+^. Further, through the analysis of the structure, they clarified that [pyr13][TFSI]-based electrolyte had higher lithium transference numbers due to the stronger interaction between [pyr13]^+^ and [TFSI]^−^. Therefore, molecular dynamics simulation has become a common tool to investigate the key structural and dynamical mechanisms. Meanwhile, it is a powerful technique for screening and designing new electrolytes in recent years.

Recently, high concentration of concentrated electrolyte (≥2 M Li salt) has attracted more and more attention of researchers. Studies indicated that high concentrated electrolytes could inhibit the formation of lithium dendrites during the lithium deposition/stripping process, thereby improving the stability of the SEI layer and the thermal stability of the electrolyte effectively (Yamada et al., 2014; Qian et al., 2015; Yamada and Yamada, 2015). More importantly, high concentrated electrolytes have an unusual solvation structure compared to conventional low concentration electrolytes. Shirai et al. (2008) investigated using Raman and NMR experiments and showed that in high concentrated electrolytes composed of LiTFSI and ILs (*N, N*-diethyl-*N*-methyl-*N*-(2-methoxyethyl)ammonium bis(trifluoromethanesulfonyl)amide, Li^+^ coordinates with four oxygen atoms within two [TFSI]^−^ anions to form the [Li(TFSI)_2_]^−^ structure. At same time, Umebayashi et al. reported the influence of temperature on the structure of high-concentration LiTFS-[C_2_mim][TFSI] electrolyte, indicating that the cis form of [TFSI]^−^ is more stable at high concentration of lithium salt (Umebayashi et al., 2008). After that, Yamada et al. reported that the performance of 3.6 mol/L electrolyte consisted of dual (fluorosulfonyl) lithium amide (LiFSA) and DME was much higher than that of commercial electrolytes under ultrafast charge. This discovery is an important breakthrough in fast-charging Li ion batteries and also broadens our knowledge that the performance of high concentration electrolyte is poor (Yamada et al., 2013). Recently, we also carried out a study on highly concentrated IL electrolytes. By comparing 2 mol/L lithium salt (LiTFSI) with pure organic solvents (DMC and DEC) and IL solvents ([C_*n*_mim][BF_4_] and [C_*n*_mim][TFSI] (*n* = 2, 4)), we found that IL electrolytes had higher conductivity than organic solvents at high concentration of Li salt; meanwhile, the dissolution of LiTFSI in the IL solvents was an anion-driven process (Tong et al., 2019). To sum up, high concentrated IL electrolytes are promising for the development of high voltage and high energy density batteries.

In summary, owing to the enormous possibilities of IL electrolytes, how to effectively screen ILs, explore the electrolyte formulation, and design new high-performance IL electrolytes has become the key part to improve the performance of lithium-ion batteries. In this work, a range of IL electrolytes including 1-alkyl-3-methyl imidazoled-based ILs ([C_*n*_mim][TFSI] and [C_*n*_mim][FSI] (*n* = 2,4)) doped with four different concentrations of lithium bis(trifluoromethylsulfonyl) imide (LiTFSI) (0.3, 0.5, 1.5, and 2.0 mol/L) are investigated. The effect of lithium concentration on the performance of IL electrolytes, such as density, viscosity, self-diffusion coefficient, lithium ion transference number, and the structures were revealed combining computational and experimental techniques.

## Materials and Methods

### Experimental

In this work, all the IL electrolytes including the lithium bis(trifluoromethanesulfonyl)imide (LiTFSI) and four different pure ILs, i.e., 1-ethyl-3-methylimidazolium bis[(trifluoromethyl)sulfonyl]imide ([C_2_mim][TFSI]), 1-ethyl-3-methylimidazolium bis(fluorosulfonyl)imide ([C_2_mim][FSI]), 1-butyl-3-methylimidazolium bis(trifluoromethylsulfonyl)imide (C_4_mim][TFSI]), and 1-butyl-3-methyl-imidazolium bis(fluorosulfonyl)imide (C_4_mim][FSI]) were purchased from Lanzhou Institute of Chemical Physics, Chinese Academy of Sciences. All the samples were obtained by mixing the ILs with the different molarity concentration (0.3, 0.5, 1.5, and 2.0 mol/L) of LiTFSI and stirring for overnight in an argon filled glovebox.

The density and viscosity measurements were carried out by using a viscosity/density meter (DMA5,000M-Lovis2,000ME) at 25°C. Furthermore, the diffusion property of electrolytes was measured as follows: ~20 mg of the samples was dispersed in 1 ml of D_2_O, and it was measured in a 5-mm NMR (nuclear magnetic resonance) tube. DOSY (diffusion ordered spectroscopy) NMR measurements were conducted on a Bruker spectrometer (500 WB AVANCE III). The instrument was equipped with a 5-mm PABBO probe (operating at 500.137 MHz for ^1^H, ^19^F, ^7^Li) and a z-gradient coil with a nominal maximum gradient of 50 G cm^−1^. The pulse sequence was a ledbpgp2s (longitudinal eddy current delay bipolar gradient pulse). Experiments were carried out with 8 scans (^1^H and ^19^F: Δ = 100 ms; ^7^Li: Δ = 100 ms). The DOSY spectra acquired on the spectrometer were processed with Bruker Topspin 3.2. All results will be discussed later in detail.

### Simulation Details

In this work, all atom MD simulations were performed for four ILs ([C_*n*_mim][TFSI], [C_*n*_mim][FSI] (*n* = 2, 4)) electrolytes at four different lithium salt LiTFSI concentrations (0.3, 0.5, 1.5, and 2 mol/L) at 298 K. The atom Li was described by the Amber force field (Wang et al., 2004). Meanwhile, all ILs were described by an optimized Amber force field developed by Liu et al. (2004) as they have shown that a reliable description of the density, diffusion coefficients, and conductivity could be achieved for ILs based on this force field. In addition, the restrained electrostatic potential (RESP) procedure was used to process the partial charge of all ILs (Bayly et al., 1993). Further, due to the polarization effect, the electrostatic interaction between ion-ion will overestimate. Thus, to solve this problem, reduce the partial charge of the atom to 0.8 times. Therefore, more accurate thermodynamic and structure properties of IL electrolytes in this work could be obtained (Maginn, 2009; Salanne, 2015). The effectiveness of this method is also reported by Schmollngruber et al. (2015).

In order to maintain different salt concentrations in this work, 100 pairs of LiTFSI were placed in periodic boundary simulation boxes with different numbers of ILs, respectively (Table 1). All simulations were performed with periodic boundary conditions in a cubic box and the initial configurations were built by Packmol package (Martínez et al., 2009). All MD simulations were employed by Gromacs software (Van Der Spoel et al., 2005). The Verlet algorithm was used to integrate Newton's equations of motion. Meanwhile, the van der Waals and electrostatic interaction were treated with the Lennard-Jones potential and the Particle Mesh Ewald (PME) algorithm, respectively. For each system, the canonical ensemble (NVT) and the isothermal isobaric ensemble (NPT) were relaxed for the first 10 ns and the next 60 ns, respectively. Furthermore, the NPT ensemble and the microcanonical ensemble (NVE) were carried out for 50 and 10 ns to achieve the configurational equilibria. In the process of simulation, the trajectory was recorded every 0.1 ps with a time step of 1.0 fs for further analysis.

**Table 1:** Compositions of simulation systems in this work.

**System**	**Number of**		**Concentration of solute**	
	**Solvent IL pairs**	**Solute LiTFSI**	**M (mol/L)**	**C% (wt%)**
LiTFSI-[C_2_mim][TFSI]	964	100	0.3	5.7
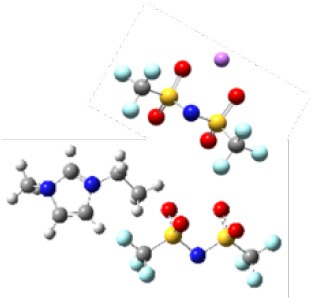	551	100	0.5	9.6
	140	100	1.5	29.5
	88	100	2.0	39.9
LiTFSI-[C_2_mim][FSI]	1,540	100	0.3	6.0
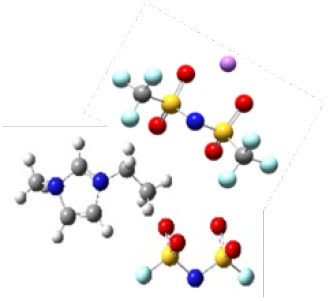	882	100	0.5	10.1
	223	100	1.5	30.6
	141	100	2.0	41.2
LiTFSI-[C_4_mim][TFSI]	900	100	0.3	6.0
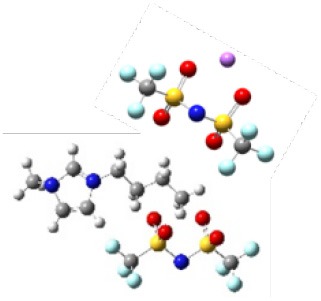	515	100	0.5	10.1
	130	100	1.5	30.8
	82	100	2.0	41.4
LiTFSI-[C_4_mim][FSI]	1,327	100	0.3	6.3
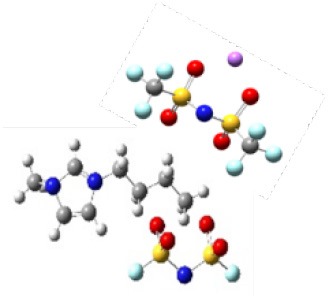	760	100	0.5	10.6
	192	100	1.5	31.9
	121	100	2.0	42.6

## Results and Discussions

### Physicochemical Properties

**Figure 2:**
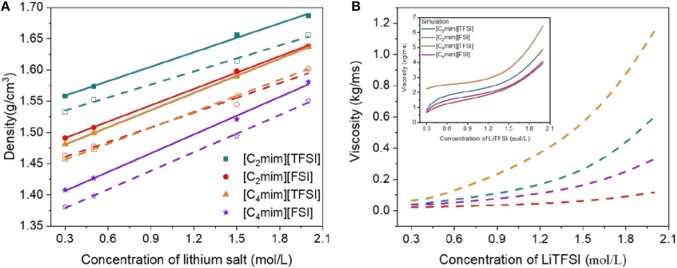
Density **(A)** and viscosity **(B)** vs. concentration of LiTFSI for all ionic liquid electrolytes (the dashed line and open points are the result from experiment and the solid line and filled points are from MD simulation).

High density and moderate viscosity for electrolytes provide more powerful energy and prevent solvent evaporation effectively. Therefore, as the energy storage capacity of batteries is improved, less environmental pollution would be formed. In this work, the density (ρ) and dynamic viscosity (η) from experiment and simulation at atmospheric pressure as a function of concentration of LiTFSI for all the four IL electrolytes were investigated (Figure 2). Clearly, the simulated density was slightly higher than the experimental value, but the errors were all <3%. Viscosity differed by an order of magnitude due to the limitation of the non-polarizable force field, but the trend remained the same. Taking into account the simplicity of the force field employed in this study, the simulation results were relatively satisfactory. Similar results have also been reported by Rey-Castro and Vega (2006).

In Figure 2, the trends of density with concentration of LiTFSI were linear; meanwhile, viscosity was almost exponential for all IL electrolytes in this work. We previously reported that due to their strong interactions between Li^+^ and TFSI^−^, adding lithium salt LiTFSI to the electrolyte led to the increase of density (Tong et al., 2019). Meanwhile, for a common cation ([C_2_mim]^+^ or [C_4_mim]^+^), [TFSI]^−^-type IL electrolytes had higher density and viscosity than [FSI]^−^-type IL electrolytes. These results were consistent with the research of Gouveia et al., who proved that when the ILs have a common cation, the densities are related to the number of denser atoms in the anions (the density of IL is higher when higher proportions of oxygen and/or fluorine atoms are present in anions) (Gouveia et al., 2017). However, for a common anion ([TFSI]^−^ or [FSI]^−^), the density of the IL electrolytes decreased as the length of the side chain increased, and the viscosity followed a reversed trend. In addition, according to the function of the viscosity and concentration of lithium salt, the viscosity of the [FSI]^−^-type IL electrolytes changed slowly compared with that of the [TFSI]^−^-type electrolytes.

### Transport Properties

The transport of ions in MD simulations is usually measured by the self-diffusion coefficient (D), which is a function of the mean square displacement (MSD) shown in Equation (1). A larger D value in a given time means a faster diffusion dynamics.

(1) $$\begin{array}{r} \text{D}=\frac{1}{6}\underset{t\to \infty }{\lim}\frac{d}{dt}\langle \sum_{i=1}^{N}{\left[\vec{r_{i}}\left(t\right)-\vec{r_{i}}\left(0\right)\right]}^{2}\rangle \end{array}$$

where $\vec{r_{i}}\left(t\right)$ indicates the positional vector of the center of mass of the *i*th ion at time *t*.

**Figure 3:**
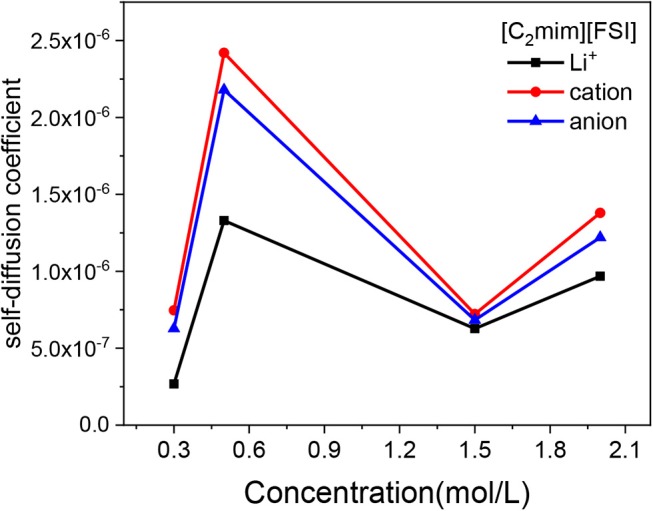
Mean square displacement (MSD) of Li^+^, [C_2_mim]^+^, and [FSI]^−^ vs. concentration of LiTFSI for LiTFSI-[C_2_mim][FSI] at 298 K.

In this work, the MSDs of Li^+^, cation, and anion of IL in electrolytes were calculated from 2 to 6 ns. Then, the slope of the MSD*-t* plots were linearly fitted, the self-diffusion coefficients could be obtained, as an example of the LiTFSI-[C_2_mim][FSI] electrolytes under four different concentrations at 298 K (Figure 3). When the concentration of lithium salt increased from 0.3 to 2 mol/L, the D of all ions showed two turning points: the ion motion rapidly increased from 0.3 to 0.5 mol/L and then dropped slowly until the concentration was above 1.5 mol/L, followed with an increase again after 1.5 mol/L. In addition, the trend in self diffusion coefficient was D_cation_ > D_anion_ > ${\text{D}}_{\text{Li}}^{+}$, which was consistent with the conclusion of Liu and Maginn (2013) in which they indicated that the small Li^+^ has the slowest diffusivity due to the fact that Li^+^ interact strongly with anion of ILs (details of the interaction of Li^+^ and TFSI^−^ is discussed in Structural Analyses). Details on the results of self-diffusivity coefficients for all IL electrolytes are shown in Table S1. More importantly, in terms of experiment, we also got the same trend through NMR measured and the results are listed in Table S2. The results of the experiments and simulations are different in several orders of magnitude due to the high polarization degree of electrolyte in this study. At present, it is very difficult to use the traditional force field to accurately calculate this system. Therefore, in future work, a polarizable force field could be optimized for transport properties.

In a given time period, lithium ion transference number *T*_*Li*_, i.e., the contribution to conductivity due to the Li^+^ transport, can be approximated from D through an equation (Lesch et al., 2016; Chen et al., 2018):

(2) $$\begin{array}{r} T_{Li}=\frac{N_{Li}D_{Li}}{\sum N_{i}D_{i}} \end{array}$$

where *N*_*i*_ is the number of ions of species *i*, and *D*_*i*_ is the corresponding self-diffusion coefficient. The resulting lithium ion transference numbers are shown in Table 2.

**Table 2:** The lithium ion transference number (*T*_*Li*_) for all IL electrolytes at four concentrations of LiTFSI.

**Concentration of LiTFSI (mol/L)**	**[C_**2**_mim] [TFSI]**	**[C_**2**_mim] [FSI]**	**[C_**4**_mim] [TFSI]**	**[C_**4**_mim] [FSI]**
0.3	0.015	0.013	0.028	0.018
0.5	0.047	0.031	0.041	0.028
1.5	0.217	0.167	0.154	0.188
2.0	0.277	0.209	0.350	0.290

Lithium transference number is one of the most important properties for lithium battery. Obviously, Table 2 shows that the transference number of lithium increased with the increase of lithium salt concentrations. In addition, the transference number of lithium ions was approximately 24% higher in [C_2_mim][TFSI] electrolyte than in [C_2_mim][FSI] electrolyte. Meanwhile, 14% higher in the [C_4_mim][TFSI] electrolytes than in [C_4_mim][FSI] for all lithium salt concentrations. Therefore, the lithium ion dynamics in the [TFSI]^−^-type systems are relatively faster in the same length of side chain of IL electrolytes system. This phenomenon is mainly due to the different interaction between Li^+^ and [TFSI]^−^ of ILs.

### Structural Analyses

The Radial Distribution Function (RDF) (Méndez-Morales et al., 2013) is a powerful method to explore the relationship between the structure and physicochemical properties on the micro scale. Therefore, the RDF for center of mass of the ions in this work was investigated:

(3) $$\begin{array}{r} g\left(r\right)=\frac{\langle \underset{i,j}{\sum }\delta \left(r-r_{ij}\right)\rangle }{N\rho }, \end{array}$$

where *N* is the number of particles, ρ is the number density, and *r*_*ij*_ is the spatial distance of the particles *i* and *j*.

The *coordination number* (*N*) (Lourenço et al., 2018) is a function of RDF, as shown in Equation (4). The number of particle *j* surrounding the particle *i* in its first solvation shell is described by the coordination number *N*_(*i*−*j*)_,

(4) $$\begin{array}{r} N_{\left(i-j\right)}=4\pi \rho_{j}\int_{0}^{r^{\prime }}g_{ij}\left(r\right)r^{2}dr, \end{array}$$

where *r*′ is the first minimum in the *g*(r) plot, *r* is the distance, and ρ_*j*_ is the density of particle *j*.

The *residence time* (τ) reflects the degree of solvation of the ILs, which is determined from the integration of the autocorrelation function (ACF). The residence time of cation and anion of ILs in the first coordination shell of Li^+^ could be calculated by Equation (5):

(5) $$\begin{array}{r} \tau_{i}=\int_{0}^{\infty }ACF\left(t\right)\\ ACF\left(t\right)=\frac{\langle B_{ij}\left(t\right)B_{ij}\left(0\right)\rangle }{\langle B_{ij}\left(0\right)B_{ij}\left(0\right)\rangle }, \end{array}$$

where *B*_*ij*_ = 1 if ions *i* and *j* are inside the first coordination shell of each other; otherwise, *B*_*ij*_ = 0.

**Figure 4:**
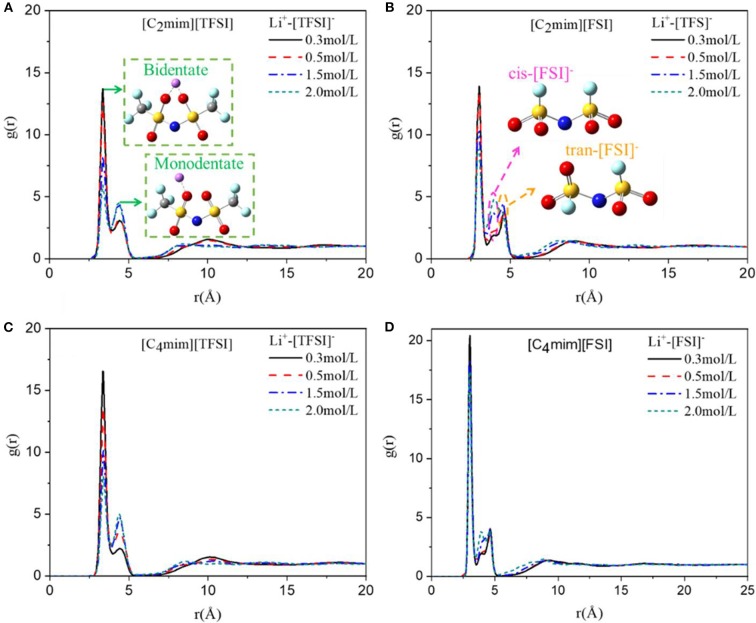
Radial distribution function of Li ion and anion of **(A)** [C_2_mim][TFSI], **(B)** [C_2_mim][FSI], **(C)** [C_4_mim][TFSI] and **(D)** [C_4_mim][FSI] at four concentrations of lithium salt LiTFSI at 298 K.

As mentioned in our previous studies, there was an anion driving effect in the IL electrolytes, i.e., lithium ions mainly interacted with anions of IL in the electrolyte solution (Tong et al., 2019). Therefore, in this section, as shown in Figure 4, we studied the effect of four lithium salt concentration on the structure and interaction of Li ions with anions in ILs by radial distribution function, coordination number, residence time, ion association trend, and solvation effect.

#### Coordination Structure

As shown in Figure 4, the RDF between lithium ion and anion of ILs in four types of IL electrolytes at all concentrations of LiTFSI was investigated. Obviously, the interaction between lithium ion and anion became weaker when lithium concentration increased. More importantly, for the same side chain, the structure of [TFSI]^−^-type IL electrolytes changed dramatically with the concentration of lithium salt compared to that of [FSI]^−^-type IL electrolytes (Figures 4A,B), which reflected the slower change of viscosity of the [FSI]^−^-type systems as mentioned above. Furthermore, the interactions for longer side chain of cation ([C_4_mim]^+^) were much stronger for the [FSI]^−^-type IL electrolytes (Figures 4B,D), which indicated why [C_4_mim] [FSI] has a higher viscosity than [C_2_mim] [FSI]. However, for the [TFSI]^−^-type electrolytes, the interaction between Li^+^ and anion of [C_4_mim] [TFSI] was enhanced comparing with that of [C_2_mim] [TFSI] when the concentration of LiTFSI was <1.5 mol/L. However, when the concentration was high (1.5–2 mol/L), they were almost the same. This indicated that the cations of imidazole-based ILs had an effect on the interaction between anions and lithium ions at low concentration of lithium salt. However, the influence of cations gradually decreased as the concentration of LiTFSI increased. Further, our simulation results revealed that the Li^+^-anion had a double-peak structure between 2.8 and 5.0 Å regardless of the type of ILs. Meanwhile, the first peak was much higher than the second peak. This indicated that a strong coordination between Li^+^ and anion in the electrolyte system existed and the structure of the first peak stability was less affected by the type of ILs. More importantly, as shown in Figure 4A, two possible coordinations of anion and lithium ion were proved by the double-peak structure, in which the first peak represented the bidentate coordinating of Li^+^ and anion (TFSI^−^ or FSI^−^), and the second one represented monodentate coordination. The same conclusions were also obtained by Lesch et al. (2016). Additionally, after the primary peak at 2.8 Å, we also observed two secondary RDF peaks at 3.5 and 4.2 Å. According to our previous research, the main reason for the two secondary peaks was the cis–trans form structure of anion where cis-FSI mainly appeared at the peak position of 3.5 Å, while trans-FSI mainly appeared at 4.2 Å (Figure 4B).

**Figure 5:**
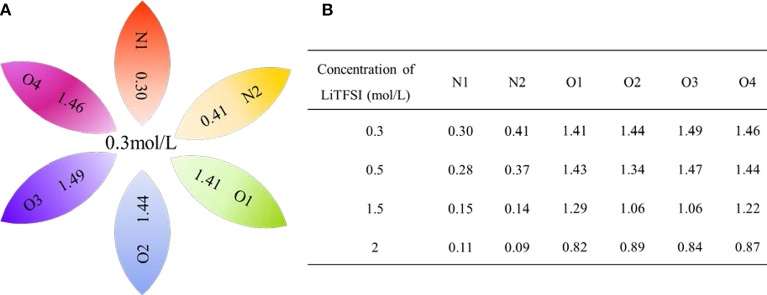
Site–site coordination number (*N*) of Li^+^-O(FSI^−^) and Li^+^-N(FSI^−^) for the LiTFSI-[C_2_mim][FSI] electrolyte system **(A)** in the 0.3 mol/L of LiTFSI **(B)** in the four different concentrations of lithium salt.

**Figure 6:**
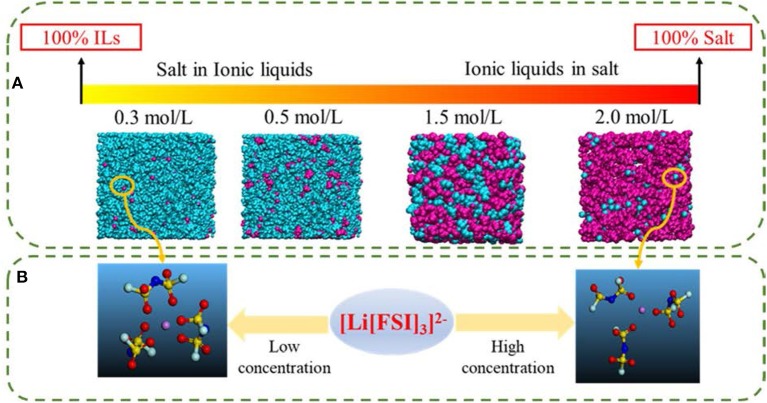
**(A)** Snapshots of four different lithium concentrations of the LiTFSI-[C_2_mim][FSI] system from MD (blue represents ionic liquids, pink represents lithium salts). **(B)** The [Li[FSI]_3_]^2−^ configurations were randomly captured at 0.3 and 2 mol/L for the LiTFSI-[C_2_mim][FSI] electrolyte system, respectively.

In order to further investigate the effect of lithium salt concentration on the ion association for IL electrolytes, in this study, the coordination of lithium ion and anion of ILs was analyzed. One example of where the coordination number of each Li^+^-O(FSI) and Li^+^-N(FSI) for LiTFSI-[C_2_mim] [FSI] system is given in Figure 5. In our previous work, we proved that lithium ions were mainly coordinated with oxygen atoms of anions in the ILs. Therefore, the sum of coordination numbers of Li^+^-O(FSI) were computed, when concentration of LiTFSI was 0.3 mol/L, N(Li-O) was 5.8; however, it was decreased to N(Li-O) = 3.42 in 2.0 mol/L LiTFSI-[C_2_mim][FSI] system. Our hypothesis about the reason was that the ionic cluster, i.e., [Li[FSI]_3_]^2−^ was formed in the electrolytes, which was validated in MD snapshots in this work and shown in Figure 6. As mentioned above, the lithium ion was located at the center of the three anions when the concentration of LiTFSI was low, because of the bidentate coordination for Li-[FSI]^−^. When the concentration of LiTFSI increased, the lithium ion and oxygen atom gradually leaned toward monodentate coordination. However, the structure of the ion cluster always consisted of three anions and one lithium ion, which was consistent with vibrational spectroscopic analysis by Lassègues et al. (2009). The coordination number of lithium ions and oxygen atoms for other IL electrolytes was also calculated and listed in Table S3. Meanwhile, the ionic cluster [Li[TFSI]_3_]^−^ was found in other LiTFSI-IL systems, and the same conclusion was also demonstrated by Monteiro et al. (2008).

#### The Residence Time

The residence time of cation and anion of ILs in the first coordination shell of Li^+^ is one of the most important properties for the stripping solvation shell in lithium ion battery. It could be calculated according to Equation (5) and shown in Table 3. Obviously, increasing the concentration of LiTFSI led to an increase of the residence time for all IL electrolytes. As mentioned above, increase in the concentration of lithium salt resulted in a more closed and compact coordination structure. Therefore, a closed coordination shell should exist due to the higher residence time. In addition, the residence times in the [C_2_mim]^+^-based electrolyte systems were lower than those in the [C_4_mim]^+^-based systems. This mainly related to the different interactions of [C_2_mim]^+^ and [C_4_mim]^+^ with Li. More importantly, strong correlation/coupling between the residence time and the mobility of Li^+^ had been proved by Li et al. (2015). Therefore, it is the main reason why the lithium ion transference numbers increase with the increase of the concentration of LiTFSI.

**Table 3:** Residence time (ns) of cation and anion for all ILs in the first coordination shell of Li^+^.

**Ionic liquids**	**0.3 mol/L**	**0.5 mol/L**	**1.5 mol/L**	**2.0 mol/L**
[C_2_mim][TFSI]-cation	3.18	4.08	4.59	4.71
[C_2_mim][TFSI]-anion	3.72	4.22	4.63	4.74
[C_2_mim][FSI]-cation	3.71	4.27	4.68	4.74
[C_2_mim][FSI]-anion	3.78	4.08	4.69	4.64
[C_4_mim][TFSI]-cation	4.14	4.284	4.658	4.743
[C_4_mim][TFSI]-anion	4.15	4.43	4.64	4.66
[C_4_mim][FSI]-cation	4.29	4.47	4.75	4.82
[C_4_mim][FSI]-anion	3.95	4.09	4.71	4.85

## Conclusions

ILs are used as electrolytes in energy storage devices due to their unique characteristics, thereby improving the safety and energy storage capacity of lithium ion batteries. In this work, we reviewed the development and research trends of IL electrolytes, and clarified the great possibilities of IL electrolytes. Combining atomistic MD simulations and fundamental physical property experiments, we investigated the effect of lithium concentration on the performance of electrolyte in four IL solvents ([C_*n*_mim][TFSI] and [C_*n*_mim][FSI], *n* = 2, 4).

The physicochemical properties of all IL solvent electrolytes were calculated and measured at the first. Simulation results showed that the density and viscosity increase with the increase of the concentration of LiTFSI for all LiTFSI-ILs electrolytes. As shown in the simulation results, higher values of both density and viscosity of LiTFSI-IL electrolytes were detected as the concentration of LiTFSI increases. The turning point of the self-diffusion coefficient indicates that the migration of ions in the IL electrolytes is non-linear with concentration. Therefore, exploring the extremes value of the electrolyte concentration environment is critical to improving the lithium ion migration and the battery performance. Later, we investigated the effect of the concentration of lithium salt on the ionic associations of the ions Li^+^ and ILs by evaluating the radial distribution function and ionic coordination number. For all IL electrolytes, a strong coordination between Li^+^ and anion of ILs, bidentate and monodentate coordinating were observed at the positions between 2.8 and 5.0 Å, respectively. Meanwhile, the cis and trans isomerism of [FSI]^−^ were observed to appear near Li^+^ at the positions of 3.5 and 4.2 Å. Further, the ionic cluster [Li[anion]_3_]^2−^ in the IL electrolytes has been found by analyzing the coordination of lithium ion and anion of ILs. Additionally, by calculating the residence time of cation and anion of ILs in the first coordination shell of Li ion, we see that increasing the concentration of LiTFSI leads to a more closed and compact coordination structure and that a strong correlation/coupling between the residence time and the mobility of Li^+^ has been demonstrated.

## Data Availability Statement

All datasets generated for this study are included in the article/Supplementary Material.

## Author Contributions

JT contributed to the design of the study, performed the simulation for electrolytes, and wrote the manuscript. SW contributed to the experimental research. All authors contributed to manuscript revision, read, and approved the submitted version.

### Conflict of Interest

The authors declare that the research was conducted in the absence of any commercial or financial relationships that could be construed as a potential conflict of interest.
