# Supplementary material for: The Effect of Concentration of Lithium Salt on the Structural and Transport Properties of Ionic Liquid-Based Electrolytes
Source: Front Chem. 2020 Feb 4;7:945. doi: 10.3389/fchem.2019.00945 (PMC7010713; doi:10.3389/fchem.2019.00945)
Supplement: Supplementary file 1 [file Presentation_1.pdf]

The effect of concentration of lithium salt on the structural and transport properties of ionic liquids-based electrolyte

Jiahuan Tong[a,b], Shengli Wu[a], Nicolas von Solms[b], Xiaodong Liang[b,*], Feng Huo[a,*], Qing Zhou[a,c], Hongyan He[a], Suojiang Zhang[a,c,*]

[a]*Beijing Key Laboratory of Ionic Liquids Clean Process, Institute of Process Engineering, Chinese Academy of Sciences, Beijing 100190, P.R. China*

[b]*Department of Chemical & Biochemical Engineering, Technical University of Denmark, DK 2800 Kgs. Lyngby, Denmark*

[c]*College of Chemistry and Chemical Engineering, University of Chinese Academy of Sciences, Beijing 100049, P. R. China*

1. Introduction

The following figure and tables contain the number of articles published in ionic liquids electrolytes in recent years and the results of self-diffusion coefficients on MD simulation and NMR experiment at 298K, respectively. Meanwhile, the result of site-site coordination number between the Li ion of lithium salt (LiTFSI) and the oxygen atom in the anion of ILs for all ILs electrolyte ([C_2mim][TFSI], [C_2mim][FSI], [C_4mim][TFSI] and [C_4mim][FSI]) electrolytes at four different concentration of lithium salts.

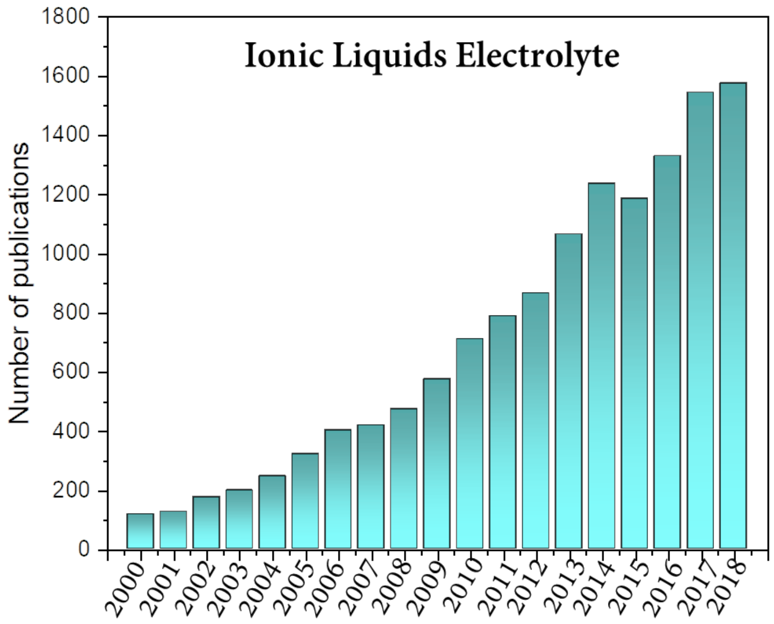

Figure S1. Number of articles published in ionic liquids electrolytes in recent years. *(Data source from Web of Science, Keywords: Ionic liquids, Electrolyte)*

Table S1. Self-diffusion coefficients (unit: 10^{-7} m^2/s) of the all ILs electrolytes by MD simulation.

$[C_2mim][TFSI]$	Li	ca	an
0.3	2.36	8.70	6.89
0.5	16.6	33.5	28.0
1.5	1.47	2.94	2.34
2.0	4.52	3.69	5.47
$[C_2mim][FSI]$			
0.3	2.68	7.46	6.28
0.5	13.3	24.2	21.8
1.5	6.27	7.22	6.83
2.0	9.68	13.8	12.2
$[C_4mim][TFSI]$			
0.3	3.95	8.23	6.85
0.5	2.85	6.80	6.20
1.5	2.74	6.55	4.99
2.0	8.69	10.5	9.19
$[C_4mim][FSI]$			
0.3	2.30	4.55	4.73
0.5	2.19	4.43	4.92
1.5	6.23	7.00	7.01
2.0	45.2	45.4	46.1

Table S2. Self-diffusion coefficients (unit: 10^{-10} m^2/s) of the all ILs electrolytes by NMR experiment.

$[C_2mim][TFSI]$	0.3	0.5	1.5	2.0
H	8.430	9.188	9.145	9.291
Li	1.845	8.973	8.672	8.731
$[C_2mim][FSI]$				
H	9.420	9.477	9.182	9.435
Li	8.689	8.769	8.481	8.618
$[C_4mim][TFSI]$				
H	7.933	7.353	6.917	7.209
Li	9.096	8.243	8.203	8.705
$[C_4mim][FSI]$				
H	7.325	7.070	7.135	7.172
Li	8.202	8.483	8.632	9.059

Table S3. Site-site coordination number (*N*) of Li^{+}-O(anion of ILs) for all ILs electrolyte systems in this work

$[C_2mim][TFSI]$	0.3	0.5	1.5	2.0
O1	1.41	1.42	1.28	0.82
O2	1.38	1.33	1.06	0.86
O3	1.45	1.46	1.05	0.89
O4	1.38	1.44	1.21	0.85
$[C_2mim][FSI]$				
O1	1.41	1.43	1.29	0.82
O2	1.44	1.34	1.06	0.89
O3	1.49	1.47	1.06	0.84
O4	1.46	1.44	1.22	0.87
$[C_4mim][TFSI]$				
O1	1.39	1.42	1.29	0.83
O2	1.39	1.34	1.06	0.85
O3	1.46	1.47	1.06	0.89
O4	1.39	1.44	1.22	0.91
$[C_4mim][FSI]$				
O1	1.40	1.41	1.27	0.85
O2	1.39	1.32	1.04	0.86
O3	1.43	1.44	1.06	0.87
O4	1.35	1.42	1.22	0.93
